# Fabrication of Highly Conductive Porous Cellulose/PEDOT:PSS Nanocomposite Paper via Post-Treatment

**DOI:** 10.3390/nano9040612

**Published:** 2019-04-13

**Authors:** Youngsang Ko, Jeonghun Kim, Dabum Kim, Goomin Kwon, Yusuke Yamauchi, Jungmok You

**Affiliations:** 1 Department of Plant & Environmental New Resources, Kyung Hee University, 1732 Deogyeong-daero, Giheung-gu, Yongin-si, Gyeonggi-do 446-701, Korea; ysko1119@naver.com (Y.K.); kdb511@naver.com (D.K.); gracegm@hanmail.net (G.K.); 2 School of Chemical Engineering & Australian Institute for Bioengineering and Nanotechnology (AIBN), The University of Queensland, Saint Lucia, Brisbane, QLD 4072, Australia; y.yamauchi@uq.edu.au

**Keywords:** nanocellulose, conductive polymer, paper electrode, conductive nanocomposite, post-treatment

## Abstract

In this paper, we report the fabrication of highly conductive poly(3,4-ethylenedioxythiophene)-poly(styrenesulfonate) (PEDOT:PSS)/cellulose nanofiber (CNF) nanocomposite paper with excellent flexibility through post-treatment with an organic solvent. The post-treated PEDOT:PSS/CNF porous nanocomposite papers showed a lower sulfur content, indicating the removal of residual PSS. The electrical conductivity of PEDOT:PSS/CNF porous nanocomposite paper was increased from 1.05 S/cm to 123.37 S/cm and 106.6 S/cm by post-treatment with dimethyl sulfoxide (DMSO) and ethylene glycol (EG), respectively. These values are outstanding in the development of electrically conductive CNF composites. Additionally, the highly conductive nanocomposite papers showed excellent bending stability during bending tests. Cyclic voltammetry (CV) showed a Faradaic redox reaction and non-Faradaic capacitance due to the redox activity of PEDOT:PSS and large surface area, respectively. Electrochemical energy storage ability was evaluated and results showed that capacitance improved after post-treatment. We believe that the highly conductive PEDOT:PSS/CNF porous nanocomposite papers with excellent flexibility described here are potential candidates for application in porous paper electrodes, flexible energy storage devices, and bioengineering sensors.

## 1. Introduction

Conductive nanocomposites with flexibility, lightness, cost-effectiveness, and good electrical properties are in high demand for application in flexible electronics, sensors, and energy harvesting/storage systems [1,2,3,4,5,6,7,8]. Poly(3,4-ethylenedioxythiophene)-poly(styrenesulfonate) (PEDOT:PSS), an aqueous-based conductive polymer nanoparticle, is considered to be a conductive material candidate due to its water-based processability, good conductivity, and excellent hybridization ability with other materials [9,10,11]. However, the PEDOT:PSS has limitations to form the 3D network pore structure with a high surface area which is attractive properties for various applications.

Poly(styrenesulfonate) (PSS) has been used for doping, stabilization, and film formation of PEDOT:PSS [12]. However, a PEDOT:PSS solution contains excess PSS to maintain stability, so a large amount of residual PSS remains after film formation. Researchers have mixed polar solvents with a PEDOT:PSS solution to form PEDOT:PSS film by drying at 100–150 °C because polar solvents help to remove excess PSS and facilitate the rearrangement of PEDOT [13,14]. In addition, various pre- and post-treatments have been developed to improve the electrical conductivity of PEDOT:PSS [12,15,16,17]. However, most research efforts have been dedicated towards improving the electrical conductivity of PEDOT:PSS film [18].

Over the last few years, cellulose-based nanocomposite materials have been widely used in various applications such as bioelectronics [19], flexible and portable devices [20], eco-friendly devices [21], and energy storage [22] because of their low cost, low density, high mechanical strength, good temperature resistance, high specific surface area, and chemical stability [23,24,25]. Among cellulose-based nanocomposites, electrically conductive cellulose nanocomposites have received much attention due to their broad range of applications [26] such as gas sensor, supercapacitor, and tissue engineering using graphene [27], polypyrrole [28], and polyaniline [29]. In particular, cellulose nanomaterials such as cellulose nanofibers (CNFs) and cellulose nanocrystals (CNCs) have been extensively incorporated with conductive materials such as conductive polymers [19,30], multi-dimensional carbons [31,32,33], and other active materials [34] by various processing methods due to the advantages induced by the cellulose nanomaterials. Fabrication of conductive polymer/cellulose nanocomposites by in- and ex-situ polymerization, coating, and vacuum filtration methods has been reported [19,35]. Despite their good flexibility and stability properties, however, there are limitations when used for electrode-based electrochemical sensing or storage device applications that require high electrical conductivity [36,37,38].

Herein, we report the fabrication of highly conductive porous nanocomposite paper using a mixed solution of water-dispersed PEDOT:PSS and CNF through a simple vacuum-filtration method. There have been studies on the development of PEDOT:PSS/CNF nanocomposites, but their electrical conductivities (1.8–45 S/cm) were low [19,39,40]. To be utilized as a conductive paper substrate, the improvement of electrical conductivity is required. We prepared homogeneously, well-mixed PEDOT:PSS/CNF porous nanocomposite paper by vacuum-filtration. Then, as a post-treatment, two types of organic solvents, dimethyl sulfoxide (DMSO) and ethylene glycol (EG), were used to remove the extra PSS as well as rearrange PEDOT segments to improve electrical conductivity. Our experiments revealed a significant enhancement in electrical conductivity due to the removal of residual PSS by organic solvent treatment. Importantly, conventional casting and dipping methods with these organic solvents do not allow efficient penetration of the solvent into the composite film. We confirmed that vacuum-filtration efficiently removed PSS due to the unique network structure of the nanoporous cellulose matrix, allowing PEDOT:PSS/CNF nanocomposite papers with high electrical conductivities to be obtained. Furthermore, we tested the electrochemical properties of the PEDOT:PSS/CNF porous nanocomposite papers and demonstrated Faradaic redox reaction and non-Faradaic capacitance from the redox activity of PEDOT:PSS and the porous structure of the paper, respectively.

## 2. Materials and Methods

### 2.1. Materials

DMSO, EG, and sodium hydroxide (NaOH) were purchased from Duksan Pure Chemicals Company Co., Ltd., Ansan, Korea. PEDOT:PSS and cellulose fibers (C6288, cotton linters) were purchased from Sigma-Aldrich (St. Louis, MO, USA) and used without further purification. Polytetrafluoroethylene (PTFE) membrane filter paper (dimension of 47 mm, pore size of 0.2 μm) was purchased from Advantec Co., Ltd., Tokyo, Japan.

### 2.2. Fabrication of cellulose nanofibers (CNFs)

Cellulose fiber (5 g) was added to a 2% NaOH solution (480 mL) and stirred for 3 h at room temperature. After washing the alkali-treated cellulose with distilled water by centrifugation, cellulose was loaded in 1 L of water. Then, high-pressure homogenization (Nano Disperser-NLM100, Ilshin Autoclave Co. Ltd., Daejeon, Korea, 25 passes at 1200 bar) was performed to obtain a well-dispersed CNF solution. The CNF dispersion solution was stored at room temperature without any aggregation of nanofibers.

### 2.3. Preparation of conductive PEDOT:PSS/CNF nanocomposite papers and their post-treatment

PEDOT:PSS/CNF nanocomposite paper was fabricated by a vacuum-filtration process. A mixture of the PEDOT:PSS dispersion (2 mL; 1 wt% in water) and CNF dispersion (5 mL; 0.4 wt% in water) was vortexed to obtain a homogeneously dispersed solution. The mixture solution was vacuum-filtrated on a PTFE membrane. When the water in the mixture solution was filtered out and a nanocomposite film was made, 2 mL of DMSO or EG was dropped onto the whole film surface (diameter: 45 mm). Then, further filtration was performed until all solvent was removed. After drying at 75 °C for 1 h, the resulting PEDOT:PSS/CNF nanocomposite film was peeled off from the PTFE membrane.

### 2.4. Characterization

Morphologies of the PEDOT:PSS/CNF nanocomposites were characterized by field emission scanning electron microscopy (FE-SEM, Hitachi, model S-4200, Carl Zeiss, model Merlin, Hitachi, Ltd., Tokyo, Japan). Sheet resistance measurements were performed using a sheet resistance tester (CMT-100S, Advanced Instrument Technology, Suwon, Korea). Sheet resistance values were calculated as the averages of measurements from several different positions. Film thickness was measured using a thickness measurement device (2109S-10, Mitutoyo, Japan) to calculate electrical conductivity. Bending stability tests were performed by periodically measuring the PEDOT:PSS/CNF nanocomposite paper bent at a bending angle of 180° (diameter: 2cm).

### 2.5. Electrochemical Measurements

Electrochemical measurements were performed using an electrochemical workstation (PGSTAT204, Metrohm Autolab, Utrecht, Netherlands). Cyclic voltammetry (CV) measurements were carried out using a three-electrode system from 0 V to 0.8 V. Ag/AgCl and platinum wire were utilized as the reference electrode and counter electrode, respectively. The commercially available coiled Pt wire was used as the working electrode and the PEDOT:PSS/CNF nanocomposite paper (6 mm × 6 mm) was clipped with it. All electrochemical measurements were conducted using 0.5 M H2SO4 as the electrolyte.

Gravimetric capacitances were calculated from CV curves using the following equation:

$$C_{g}=\frac{1}{ms\left(V_{f}-V_{i}\right)}\int_{V_{i}}^{V_{f}}I\left(V\right)dV$$

where *C_g_* is the gravimetric capacitance (F g^−1^), *s* is the potential scan rate, *V* is the potential with initial and final values of *V_i_* and *V_f_*, respectively, *I* is the current (A), and *m* is the mass of active material in grams.

Galvanostatic charge–discharge (GCD) measurements were carried out at varying current densities of 1, 2, 4, 6, and 10 A g^−1^. Gravimetric capacitances were calculated from the GCD curves using the following equation [41,42,43]:

$$C_{g}=\frac{I\times \int^{\text{​}}V\;dt}{M\times \Delta V^{2}}$$

where *C_g_* represents the gravimetric capacitance (F g^−1^), Δ*V* represents the potential window, *I* represents the current (A), *t* represents the discharge time (s), and *M* represents the total mass of active material (g).

## 3. Results and Discussion

**Figure 1:**
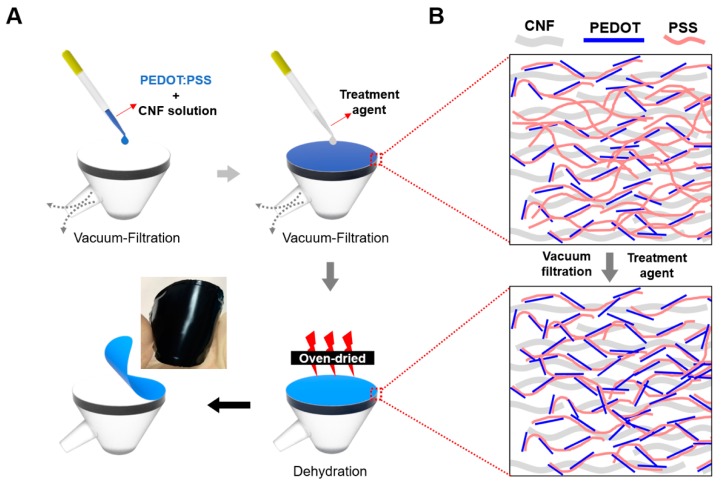
(**A**) Schematic illustration of the fabrication of post-treated poly(3,4-ethylenedioxythiophene)-poly(styrenesulfonate) (PEDOT:PSS)/CNF composite paper using a vacuum-filtration process with different treatment agents. (**B**) Morphological changes in the PEDOT:PSS/cellulose-nanofiber porous composite film through post-treatment involving the addition of organic solvents.

In this study, we report the fabrication of highly conductive and flexible nanocomposite paper (PEDOT:PSS/CNF) via vacuum filtration and a simple post-treatment process. Use of the conductive polymer, PEDOT:PSS, which forms a stable colloid in aqueous solution, allowed us to conduct facile aqueous solution-based processing to fabricate redox-active PEDOT:PSS/CNF porous nanocomposites with electrical conductivity. Nanocomposite paper was easily fabricated by the simple mixing of PEDOT:PSS and CNF solutions. The mixture of PEDOT:PSS/CNF appeared to be stably dispersed, as expected, due to the hydrophilic nature of CNF and PEDOT:PSS nanoparticles. As shown in Figure 1A, the dispersion of PEDOT:PSS and CNF was vacuum-filtrated on a PTFE membrane. After homogeneous PEDOT:PSS/CNF film was fabricated, a polar organic solvent, either DMSO or EG, was dropped on the PEDOT:PSS/CNF film surface and further filtration was performed. Polar organic solvents are known to enhance the electrical conductivity of PEDOT:PSS by removing PSS and facilitating the rearrangement of PEDOT segments. Based on this fact, we assumed that residual PSS, which hampers electrical conductivity, could be efficiently removed from a porous PEDOT:PSS/CNF structure by vacuum filtration with organic solvents. In addition, we reasoned that when residual PSS surrounding the PEDOT was removed, the PEDOT might structurally rearrange in the porous CNF film, leading to improved conductivity, as shown in Figure 1B.

After vacuum filtration with organic solvents (DMSO or EG) as a post-treatment, the nanocomposite film was dried in an oven and easily peeled off of the PTFE membrane. The prepared PEDOT:PSS/CNF porous nanocomposite paper showed a homogeneous morphology and was flexible, as shown in the photograph in Figure 1A.

**Figure 2:**
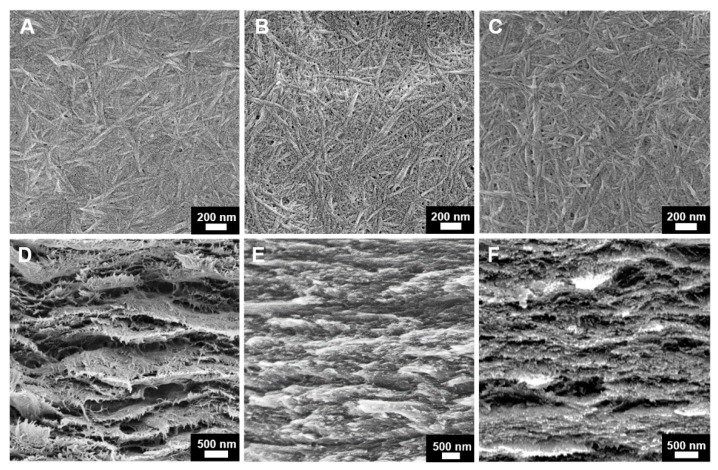
SEM images of (**A**–**C**) top surface and (**D**–**F**) cross section of PEDOT:PSS/CNF porous composite paper. (**A**,**D**) Pristine, (**B**,**E**) post-treatment with DMSO, and (**C**,**F**) post-treatment with EG.

The morphologies of PEDOT:PSS/CNF porous nanocomposite papers with and without post-treatment were studied by SEM. As seen from the top surface SEM images (Figure 2A–C), PEDOT:PSS/CNF with DMSO or EG post-treatment showed structural rearrangement with more pore formation than the pristine PEDOT:PSS/CNF composite film, probably due to the removal of PSS. This can be seen more clearly in the cross-sectional morphologies of the PEDOT:PSS/CNF nanocomposites shown in Figure 2D–F. Post-treated PEDOT:PSS/CNF porous nanocomposite films had a clearer layer structure than pristine film. These findings confirm that vacuum-filtering with a small amount of organic solvent can affect the morphology of the entire nanocomposite film.

**Figure 3:**
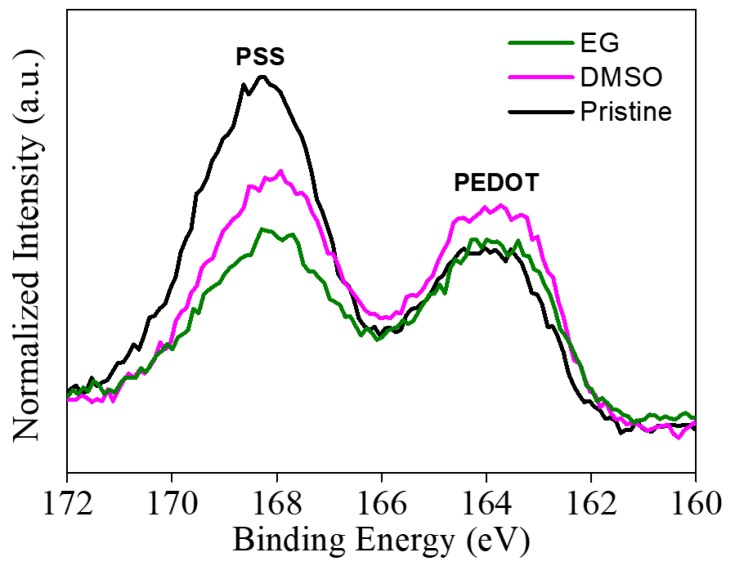
XPS S(2p) spectra of PEDOT:PSS/CNF nanocomposite paper before and after post-treatment with EG and DMSO.

We further verified the removal of residual PSS by X-ray photoelectron spectroscopy (XPS) S(2p) spectra. These spectra showed the presence of the sulfur of PSS and heterocyclic thiophene at 167.6 eV and 163.9 eV, respectively (Figure 3) [44]. PEDOT:PSS/CNF porous nanocomposite films post-treated with an organic solvent showed a significantly lower sulfur peak in the PSS region compared to the PEDOT:PSS/CNF film not subjected to post-treatment. This indicated that the residual PSS of the PEDOT:PSS/CNF nanocomposites was largely removed by the organic solvent, which might cause rearrangement of the PEDOT segments [18].

**Figure 4:**
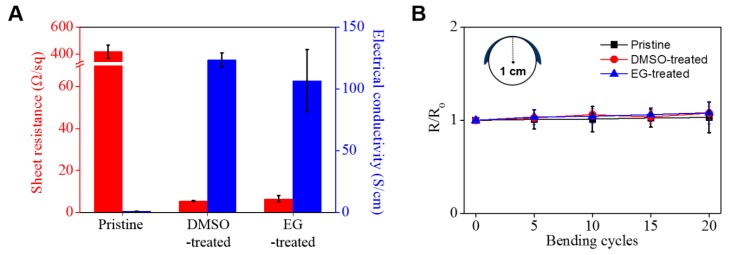
(**A**) Sheet resistance, electrical conductivity, and (**B**) bending stability of PEDOT:PSS/CNF porous nanocomposite paper before and after post-treatment with EG and DMSO.

We examined the electrical properties of the nanocomposite films by evaluating the sheet resistance and calculating the electrical conductivity (Figure 4A). PEDOT:PSS/CNF nanocomposites post-treated with either DMSO or EG exhibited a low sheet resistance of 5.41 ± 0.26 and 6.49 ± 1.49 Ω/sq, respectively, while pristine PEDOT:PSS/CNF without post-treatment had a high sheet resistance of 418.73 ± 48.94 Ω/sq (Table S1). This nearly 70-fold difference in sheet resistance indicates efficient removal of the residual PSS from PEDOT and the rearrangement of the PEDOT segments by post-treatment. Interestingly, the removal of PSS and rearrangement of PEDOT segments resulted in thickness differences. The thickness of PEDOT:PSS/CNF subjected to post-treatment was about 15 µm, while that of PEDOT:PSS/CNF without post-treatment was 23 µm. Based on these results, electrical conductivity was calculated to be 123.37, 106.6, and 1.05 S/cm for DMSO, EG, and pristine PEDOT:PSS/CNF, respectively (Figure 4A, Table S1), with the first two values being much higher than those previously reported for cellulose-based conductive polymer composites (Table S2) [45,46,47,48]. In particular, compared to the electrical conductivity values of previous studies [19,39,40] of the same materials, it increased approximately 2.7 to 68.5 times.

It is important to determine the flexibility of PEDOT:PSS/CNF nanocomposites if they are to be used as flexible conductive paper electrodes. As shown in Figure 4B, the change in sheet resistance in response to bending was evaluated for nanocomposite paper samples with a 2 cm diameter. After 20 bending cycles, the pristine PEDOT:PSS/CNF porous nanocomposite paper samples showed good flexibility due to the flexibility of CNF and the good electrical conductivity of PEDOT:PSS. In addition, the post-treated composite paper samples also exhibited similar resistance changes, indicating maintenance of the composite structure between CNF and PEDOT:PSS despite post-treatment with a polar organic solvent.

To further examine the merits of the simple vacuum-filtration method, we fabricated PEDOT:PSS/CNF porous nanocomposite paper samples and post-treated them with DMSO up to three times. There was almost no thickness difference in the samples, and the surface resistance values after one, two, and three filtration treatments were 5.41 ± 0.26, 5.25 ± 0.12, and 5.09 ± 0.12 Ω/sq, respectively (Table S3). These results demonstrate that the post-treatment of porous paper nanocomposites by the addition of a small amount of organic solvent and vacuum-filtration is an efficient method to remove residual PSS and improve electrical conductivity.

**Figure 5:**
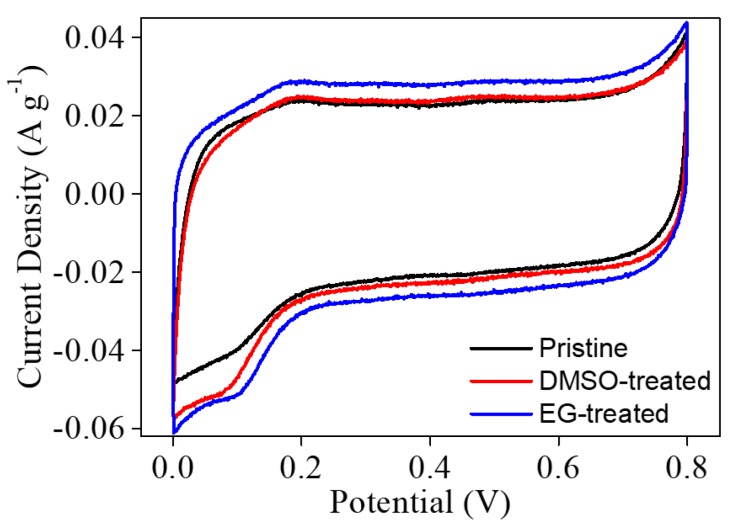
Cyclic voltammetry (CV) graphs of PEDOT:PSS/CNF composite paper samples before and after post-treatment with EG and DMSO at a scan rate of 2 mV s^−1^.

**Figure 6:**
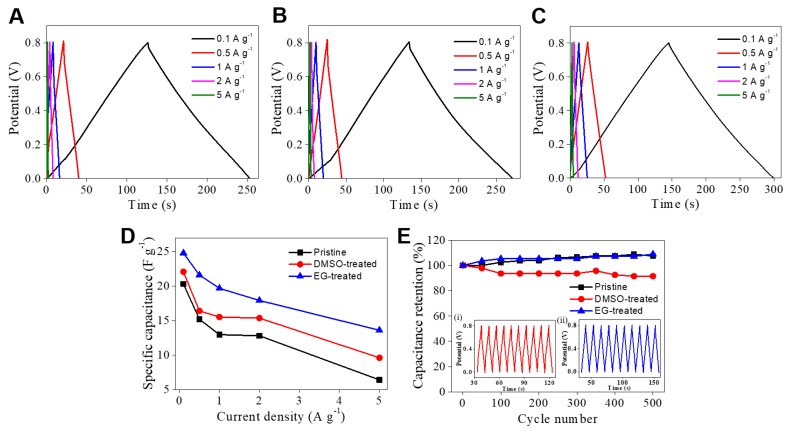
(**A**–**C**) Galvanostatic charge–discharge (GCD) curves of PEDOT:PSS/CNF composite paper samples treated with (**B**) DMSO and (**C**) EG or (**A**) without solvent post-treatment. The mass of all composite paper samples was 1.0 mg. (**D**) Current density-dependent specific capacitance of PEDOT:PSS/CNF composite paper samples. (**E**) Cycle stability of PEDOT:PSS/CNF composite paper samples at 2 A g^−1^. The inset shows 10 cycles of charge–discharge curves of PEDOT:PSS/CNF composite paper post-treated with (i) DMSO or (ii) EG.

We expected the PEDOT:PSS/CNF nanocomposites post-treated with organic solvent to have good electrochemical activity due to the redox-active PEDOT:PSS [49]. To evaluate the electrochemical activity of PEDOT:PSS/CNF porous nanocomposites, a three-electrode system with 0.5 M H_2_SO_4_ as the electrolyte was used. CV measurements were conducted within the suitable potential window of 0–0.8 V (Figure 5 and Figure S1). CV curves showed a Faradaic redox reaction and non-Faradaic capacitance from the redox activity of PEDOT:PSS (Figure 5). The peaks are observed with a shift towards the positive potential region. This might be because PEDOT:PSS/CNF composite papers with nanoporous structure cause the shift in CV response, which is consistent with the electrochemical result of the PEDOT nanostructure [50]. Interestingly, the PEDOT:PSS/CNF porous nanocomposites post-treated with EG and DMSO showed increased currents, which might be due to their increased surface area after post-treatment with polar solvents. These results were consistent with the XPS results showing the removal of PSS. The specific capacitance values of pristine, EG-, and DMSO-treated PEDOT:PSS/CNF nanocomposites at a scan rate of 2 mV s^−1^ were 45.3, 56.9, and 48.3 F g^−1^, respectively. Additionally, it was confirmed that the capacitance values were improved when compared to those of previous studies (6.21–7.4 F g^−1^) on electrically conductive CNF-based nanocomposites [39,51]. The EG post-treated PEDOT:PSS/CNF nanocomposite exhibited the highest specific capacitance at all scan rates. Galvanostatic charge–discharge curves were obtained to evaluate electrochemical energy storage ability, as shown in Figure 6. Capacitances were calculated at different current densities. Post-treated PEDOT:PSS/CNF nanocomposite films showed improved capacitances of 25 F g^−1^ (EG) and 22 F g^−1^ (DMSO) compared to the capacitance of 20 F g^−1^ of pristine film at a current density of 0.1 A g^−1^, and the PEDOT:PSS/CNF nanocomposite film post-treated with EG showed the highest capacitance, consistent with the CV results. This can be explained by efficient PSS removal and high electrical conductivity due to the rearrangement of PEDOT. In addition, the removal of PSS can help expose a redox-active PEDOT to an electrolyte. In cyclability tests up to 500 cycles, all PEDOT:PSS/CNF porous nanocomposite films showed good cyclability because of well-dispersed PEDOT:PSS in a stable, porous CNF structure.

## 4. Conclusions

In this work, we demonstrated the creation of highly conductive PEDOT:PSS/CNF porous nanocomposite paper with excellent flexibility via a simple vacuum-filtration method with organic solvent treatment. Electrical conductivities of PEDOT:PSS/CNF porous nanocomposite paper samples post-treated with DMSO and EG were significantly increased to 123.37 ± 5.87 and 106.6 ± 25.16 S/cm, respectively. These values are more than 100 times higher than that of pristine PEDOT:PSS/CNF. SEM images and XPS analysis demonstrated the removal of residual PSS as well as the structural rearrangement of PEDOT segments. CV revealed the redox activity of the resultant PEDOT:PSS/CNF porous nanocomposite paper samples. We observed improvements in capacitance after post-treatment and excellent capacitance retention after 500 charge–discharge cycles, indicating that the nanocomposite paper described here may be used in applications requiring electrochemical energy storage ability. We conclude that the two steps, vacuum filtration of the PEDOT:PSS-CNF suspension and addition of organic solvent, can produce high-performance PEDOT:PSS/CNF porous nanocomposite paper that is lightweight, portable, flexible, highly electrically conductive, and has good capacitance. PEDOT:PSS/CNF porous composite paper is a promising material for various applications such as porous paper electrodes, flexible energy storage devices, and bioengineering sensors.

## Supplementary Materials

The following are available online at https://www.mdpi.com/2079-4991/9/4/612/s1, Table S1: The electrical properties of PEDOT:PSS/CNF paper with or without the solvent post-treatment., Table S2: A comparison of conductive polymer/cellulose nanocomposites, Table S3: Sheet resistance according to the number of times DMSO filtering was conducted, Figure S1: CV graph of PEDOT:PSS/CNF composite papers.
