# Supplementary material for: Fabrication of Highly Conductive Porous Cellulose/PEDOT:PSS Nanocomposite Paper via Post-Treatment
Source: Nanomaterials (Basel). 2019 Apr 13;9(4):612. doi: 10.3390/nano9040612 (PMC6523742; doi:10.3390/nano9040612)
Supplement: Supplementary file 1 [file nanomaterials-09-00612-s001.pdf]

Supporting Information: Fabrication of Highly Conductive Porous Cellulose/PEDOT:PSS Nanocomposite Paper via Post-Treatment

Youngsang Ko[1], Jeonghun Kim*[2], Dabum Kim[1], Goomin Kwon[1], Yusuke Yamauchi[2] and Jungmok You*[1]

[1] Department of Plant & Environmental New Resources, Kyung Hee University, 1732 Deogyeong-daero, Giheung-gu, Yongin-si, Gyeonggi-do 446-701, South Korea.

[2] School of Chemical Engineering & Australian Institute for Bioengineering and Nanotechnology (AIBN), The University of Queensland, QLD 4072, Australia.

* Correspondence: jeonghun.kim@uq.edu.au; jmyou@khu.ac.kr

Table 1. Sheet resistance, thickness, and electrical conductivity of PEDOT:PSS/cellulose-nanofiber composite paper with or without the solvent post-treatment.

**Treatment agent**	**Sheet resistance (Ω/sq)**	**Thickness (μm)**	**Conductivity (S/cm)**
Pristine	418.73 ± 48.94	23	1.05 ± 0.12
DMSO	5.41 ± 0.26	15	123.37 ± 5.87
EG	6.49 ± 1.49	15	106.6 ± 25.16

*DMSO- Dimethyl sulfoxide

*EG- Ethylene glycol

Table S2. A comparison of conductive polymer/cellulose nanocomposites

Serial #	Sample	Method	Conductance (S/cm)	References
1	PANi/BC	In-situ polymerization	1.4×10^{-1}	45
2	PPy/BC	In-situ polymerization	3.39	46
3	PPy/CNF	Vacuum filtration	13.45	39
4	PPy/PEDOT:PSS/CNF	Vacuum filtration	10.55	39
5	PEDOT:PSS/BC	Ex-situ incorporation	12.17	47
6	PEDOT:PSS/Cellulose	In-situ polymerization	30	48
7	PEDOT:PSS/CNF	Vacuum filtration	2.58	39
8	PEDOT:PSS/CNF	Vacuum filtration	22.6	19
9	PEDOT:PSS/CNF	Drop-casting	45	40
10	PEDOT:PSS/CNF	Vacuum filtration	123.4	Present work

*BC- Bacterial cellulose
*PANi- Polyaniline
*PPy- Polypyrrole
*CNF- Cellulose nanofiber
*PEDOT:PSS- Poly(3,4-ethylenedioxythiophene) polystyrene sulfonate

Table S3. Sheet resistance according to the number of times DMSO filtering was conducted on the PEDOT:PSS/cellulose-nanofiber composite paper.

Number	Sheet resistance (Ω/sq)
1	5.41 ± 0.26
2	5.25 ± 0.12
3	5.09 ± 0.12

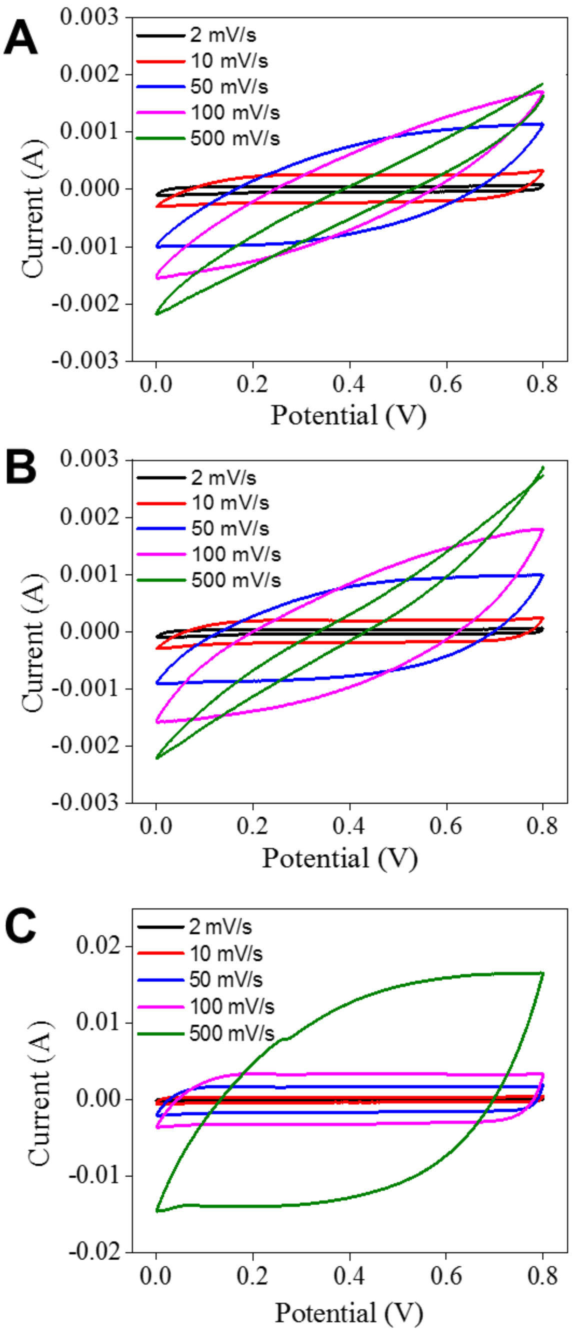

Figure S1. (A–C) Cyclic voltammetry (CV) of PEDOT:PSS/cellulose–nanofiber composite papers. (**A**) Pristine, (**B**) DMSO, and (**C**) EG.
